# Body Size Is a Significant Predictor of Congruency in Species Richness Patterns: A Meta-Analysis of Aquatic Studies

**DOI:** 10.1371/journal.pone.0057019

**Published:** 2013-02-26

**Authors:** Katherine Velghe, Irene Gregory-Eaves

**Affiliations:** 1 Department of Biology, McGill University, Montreal, Quebec, Canada; 2 Groupe de Recherche Interuniversitaire en Limnologie et en Environnement Aquatique (GRIL), Montreal, Quebec, Canada; National Institute of Water & Atmospheric Research, New Zealand

## Abstract

Biodiversity losses over the next century are predicted to result in alterations of ecosystem functions that are on par with other major drivers of global change. Given the seriousness of this issue, there is a need to effectively monitor global biodiversity. Because performing biodiversity censuses of all taxonomic groups is prohibitively costly, indicator groups have been studied to estimate the biodiversity of different taxonomic groups. Quantifying cross-taxon congruence is a method of evaluating the assumption that the diversity of one taxonomic group can be used to predict the diversity of another. To improve the predictive ability of cross-taxon congruence in aquatic ecosystems, we evaluated whether body size, measured as the ratio of average body length between organismal groups, is a significant predictor of their cross-taxon biodiversity congruence. To test this hypothesis, we searched the published literature and screened for studies that used species richness correlations as their metric of cross-taxon congruence. We extracted 96 correlation coefficients from 16 studies, which encompassed 784 inland water bodies. With these correlation coefficients, we conducted a categorical meta-analysis, grouping data based on the body size ratio of organisms. Our results showed that cross-taxon congruence is variable among sites and between different groups (r values ranging between −0.53 to 0.88). In addition, our quantitative meta-analysis demonstrated that organisms most similar in body size showed stronger species richness correlations than organisms which differed increasingly in size (r_adj_^2^ = 0.94, p = 0.02). We propose that future studies applying biodiversity indicators in aquatic ecosystems consider functional traits such as body size, so as to increase their success at predicting the biodiversity of taxonomic groups where cost-effective conservation tools are needed.

## Introduction

Biodiversity declines are so common that they are now considered to be a form of global environmental change [1], [2]. As such, scientists have been motivated to identify what factors contribute to the origin and maintenance of diversity [3], which ecosystem functions are affected by changes in diversity [4] and how best to monitor these changes [5]. Of all ecosystems, freshwaters appear to be among the most vulnerable to biodiversity losses [6], [7], [8].

The need for expediency in the protection of aquatic ecosystems has led to the use of biological indicator taxa as surrogate measures of the overall status of ecosystems (e.g. [9]). Biodiversity indicators are used because they reduce the costs required for inventories of whole communities (e.g. [10]). Among the plethora of biological indicators found in the scientific literature lie those whose aim is to predict the biodiversity of other taxonomic groups. The ability of a particular indicator group to predict the diversity of another is most often calculated as either a metric of correlation between univariate biodiversity metrics (taxonomic richness, Shannon-Weiner or taxonomic distinctness; (e.g. [11])) or as metrics of multivariate similarity of entire communities (Mantel tests on dissimilarity matrices, Procrustes analyses of ordination site scores; (e.g. [12])). Comparing the biodiversity of one taxonomic group to that of another taxonomic group is called cross-taxon congruence. A few papers have suggested that the ability of one taxonomic group to predict the community structure of another depends upon their similarity in responses to various abiotic conditions, their trophic levels, their shared evolutionary histories and their species-energy relationships [13], [14]. Interestingly, many of the factors that have been associated with the prediction of cross-taxon congruence analyses are also related to body size.

Body size has been shown to influence many characteristics of organisms [15] as it is inherently linked to lifespan [16], reproductive rate [17], trophic level [18], [19], biodiversity [20], abundance [21], density [22] and other life history traits [15], [23], [24]. Different body sizes also dictate how aquatic organisms interact with the external environment in terms of gravity, viscosity, inertia and surface tension [25] and affects the spatial scale at which physical processes can control biodiversity (e.g. local vs. regional, [26]). Recently, a few studies have speculated that body size could be considered to be an important determinant in the success of biodiversity indicators [13], [27], [28] although no formal analyses have yet addressed this assumption. Because body size influences so many patterns and processes in ecological communities, we hypothesize that body size is a significant predictor of the strength of congruency between species richness patterns. To address this hypothesis, we performed a meta-analysis of aquatic data from the published literature.

## Materials and Methods

### Identification of Studies

We initially gathered studies for our meta-analysis from a qualitative review of cross-taxon congruence studies provided by Heino [13] which reported the correlation in diversity between various taxonomic groups. This list was further supplemented with studies found using *ISI Web of Science©* and *Google Scholar* search engines (last searched January 2013) for papers containing any combination of the following keywords: (1) “biodiversity” or “species richness” and (2) “correl*”, “cross-tax*”, “congruen*” or “concordan*” and (3) “aquatic” (whereby the asterisk denotes an unconstrained search for multiple suffixes). From each study we extracted correlation coefficients, the number of study sites and the identity of organisms that were compared. We did not include correlation analyses from studies that used macrophytes or macroalgae as biodiversity indicators because the body sizes of these organisms are highly variable within and across species. We used species richness as our index of biodiversity as opposed to a multivariate metric of concordance because the former was more commonly reported in the literature. Furthermore, multivariate metrics of concordance were not consistent across studies, making them incomparable (e.g. Euclidian vs. Bray-Curtis dissimilarity matrices followed by Mantel tests, Procrustes analyses on PCA or CCA axis 1 scores). Therefore, we quantified in this study the response of alpha (local scale) diversity as opposed to beta (turnover) or gamma (landscape scale) diversity.

### Body Size Estimates

In order to investigate the effect of differences in body size on the strength of species richness concordances, we estimated the relative body size of organisms. Length was used as our measurement of body size as it is commonly used in ecological analyses of size (e.g. [29]), is readily available from classical references and is correlated to other surrogate measures of size (e.g. mass [30], [31], [32]). First, we surveyed the published literature to determine the length of each taxonomic group, on an order of magnitude scale (Table 1). We then proceeded to calculate the body size ratios between taxonomic groups. For example, the body size ratio between fish (order of magnitude length, 10 cm = 0.1 m) and macroinvertebrates (order of magnitude length, 1 mm = 0.001 m) is 0.1 m : 0.001 m = 1∶100, indicating that fish are on average 100 times larger than macroinvertebrates. We chose to conduct our analyses using an order of magnitude body size index because there is considerable variability within any one taxonomic group and because previous studies comparing body sizes have used this type of measurement (e.g. [39]). We grouped body size ratios of 1∶1000 and 1∶10 000 together in analyses to improve the sample size of that category.

**Table 1:** Order of magnitude length of organisms.

Taxa	Size (m)	Reference
Bacteria, bacterioplankton	10^−6^	Clifford 1991 [33]
Algae, phytoplankton	10^−5^	Clifford 1991
Diatoms	10^−5^	Krammer 1986–1991 [34]
Chydorids (*Chydoridae*)	10^−4^	Pennak 1989 [35]
Planktonic crustaceans	10^−4^	Pennak 1989
Planktonic rotifers	10^−4^	Pennak 1989
Zooplankton	10^−4^	Clifford 1991
Chironomids (*Chironomidae*)	10^−3^	Clifford 1991
*Heteroptera*	10^−3^	Clifford 1991
Macroinvertebrates	10^−3^	Townsend et al. 2008 [36]
Beetles (*Coleoptera*)	10^−2^	Clifford 1991
Caddisflies (*Trichoptera*)	10^−2^	Clifford 1991
Crayfish (*Astacoidea*)	10^−2^	Pennak 1989
Dragonflies (*Odonata*)	10^−2^	Clifford 1991
Gastropods (*Gastropoda*)	10^−2^	Pennak 1989
Mayflies (*Ephemeroptera*)	10^−2^	Clifford 1991
Molluscs (*Mollusca*)	10^−2^	Pennak 1989
Stoneflies (*Plecoptera*)	10^−2^	Clifford 1991
Amphibians	10^−2^	King and Behler [37]
Fish	10^−1^	Holm et al. 2009 [38]

Names and size of organisms used in studies testing species richness cross-taxon congruence in aquatic ecosystems. Names refer specifically to those employed in the text of the studies included in the meta-analysis. References for body size estimates (orders of magnitude) are included here.

### Meta-analysis Calculations

A meta-analysis is a statistical method used to quantify a general effect reported in the literature by synthesizing results across numerous studies. The statistical procedure used in meta-analysis accounts for varying degrees of reliability across individual studies by weighting the effect size from any one study by its sample size [40]. The practicality of meta-analyses has sometimes been questioned in the past because they overlook peculiarities of individual studies [41], but as was emphasized by Hillebrand and Cardinale [42], “the goal of meta-analyses is to reveal pattern and process of the whole forest, not to show what’s happening on the individual trees”. Certainly, meta-analyses have been proven to be useful in quantifying general ecological relationships such as those between species richness and ecosystem functioning [43], [44] and in identifying which factors influence the strength of trophic cascades [45], [46].

In our study, we performed a meta-analysis to compute the strength of cross-taxon congruence across groups varying in body size ratios. The effect size was measured as the Fisher’s z-transformation, which was calculated using the meta-analytic “MAc” library [47] in R statistical software [48]. The effect size is computed based on correlation coefficients (*r*) and sample sizes (n) of cross-taxon congruence presented in the literature. Confidence intervals (95%) were computed for each effect size, allowing us to determine if the effect size should be considered significant (i.e. significant when 95% confidence intervals do not overlap zero). Effect sizes based on correlation coefficients are conventionally considered to be large when they are greater than 0.4, medium when equal to 0.25 and small when inferior to 0.1 [49]. The effect sizes were subdivided according to the ratio in body sizes of the two groups that were included in each correlation analysis. For our analysis, we used all correlations reported in each study. Rosenthal’s fail-safe number (i.e. the number of studies with an effect size of zero that would be needed to render results non-significant; [50]) was also computed. To test our hypothesis, we calculated the correlation coefficient between the effect size for cross-taxon congruence of different groups and their respective body size ratios.

Because we found that sample sizes differed across body size groupings, whereby the effect sizes of smaller body size ratios were computed using more data points, we statistically reduced the sample sizes of each of the groups. Specifically, we randomly selected n = 7 correlation coefficients (i.e. the smallest number of correlation coefficients in body size groupings) from each group and reran the analysis 9 999 times using the jackknife resampling technique. We present the average from this resampling exercise. Finally, we addressed whether we violated assumptions of non-independence when several correlation coefficients from the same study were used [51]. To test for non-independence, we computed the interclass coefficient of correlation coefficients (ICC, using ANOVA framework to account for uneven group sizes; [52]), using each study as a group (e.g. [53]).

## Results

**Figure 1:**
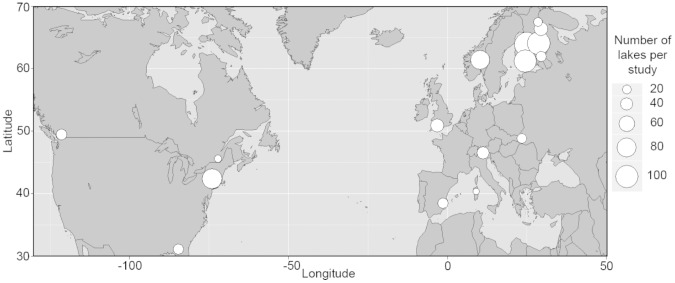
Map of studies used in meta-analysis. Size of circle refers to the number of bodies of water (lakes, ponds, streams) used to test for cross-taxon congruence in species richness in each study.

**Figure 2:**
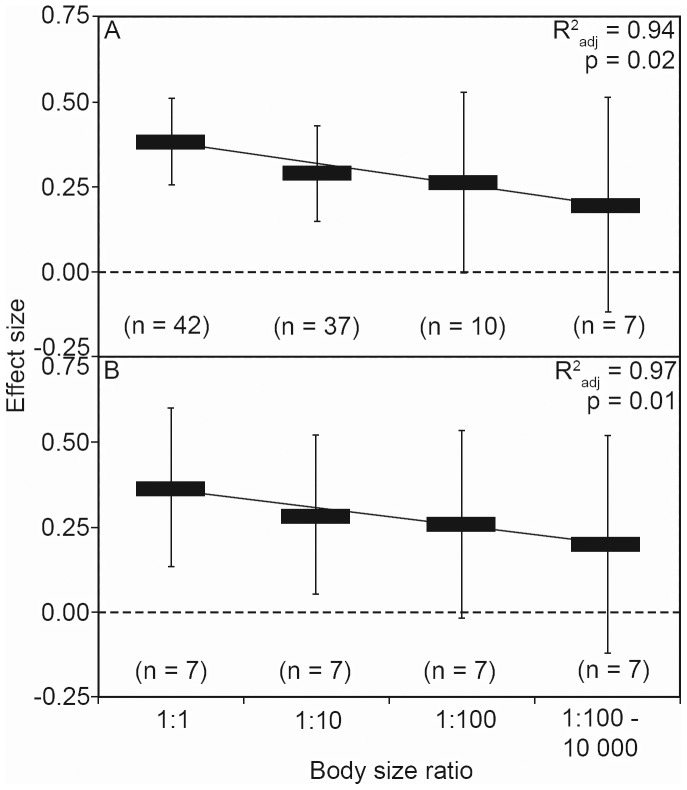
Regression analysis quantifying the relationship between the effect size for cross-taxon congruence and the ratio of body sizes of the groups being compared. The effect sizes, with associated 95% confidence intervals, for all studies found in the published literature is shown in (A) and the average effect sizes from the statistically-reduced and resampled analyses (to account for differences in sample size between body size ratios) in shown in (B). Effect sizes are significant where confidence intervals do not overlap zero.

Our literature search yielded 16 studies from across North America and Europe (Fig. 1) that fit our inclusion criteria for the meta-analysis (Table 2). We also identified a substantial number of additional studies that quantified cross-taxon congruence, but unfortunately these studies used only macrophyte or macroalgae (n = 2), or employed metrics other than species richness correlations (i.e. Mantel tests on several different dissimilarity indices or Procrustes analyses of ordination scores, n = 16). Because there was limited replication with any of these alternative analytical approaches, we had to exclude this body of literature from our study. Nonetheless, among the published studies that used species richness as their metric, we obtained 96 correlation coefficients (r range from −0.53 to 0.88) for our meta-analysis. Overall, this study thus encompassed taxonomic richness data from 784 lakes, streams and wetlands (Fig. 2). Given that the intraclass correlations value was low (ICC = 0.09, p>0.1), we were able to use all of the results reported within each study.

**Table 2:** Studies used in meta-analysis.

Study	Organisms	R
Allen et al. 1999 [26]	Benthic macroinvertebrates, fish, planktonic crustaceans, planktonicrotifers and sedimentary diatoms	−0.01–0.37
Heino 2002 [54]	Beetles, fish, dragonflies and stoneflies	−0.46–0.81
Heino et al. 2003 [12]	Caddisflies, chironomids, mayflies and stoneflies,	0.06–0.29
Heino et al. 2005 [55]	Fish and macroinvertebrates	0.26
Tolonen et al. 2005 [56]	Benthic macroinvertebrates, fish, phytoplankton and zooplankton	0.02–0.50
Sanchez-Fernandez et al. 2006 [11]	Beetles, heteropterans, mayflies, molluscs and stoneflies	−0.53–0.88
Bilton et al. 2006 [57]	Beetles, chironomids, caddisflies, and gastropods	−0.28–0.80
Longmuir et al. 2007 [58]	Bacteria, plankton and zooplankton	0–0.14
Heino et al. 2009a [59]	Diatoms and macroinvertebrates	0.51
Heino et al. 2009b [60]	Caddisflies, chironomids, mayflies, molluscs and stoneflies	0.28–0.58
Bagella et al. 2011 [61]	Beetles and crustaceans	0.16
Nascimbene et al. 2011 [62]	Algae and diatoms	−0.41
Tornblom et al. 2011 [63]	Caddisflies, mayflies and stoneflies	0.41–0.73
Korhonen et al. 2011 [64]	Bacterioplankton, phytoplankton, zooplankton	0.02–0.27
Velghe 2012 [65]	Diatoms, chydorids, macroinvertebrates and fish	0.1–0.62
Kirkman et al. 2012 [66]	Amphibians and beetles	0.21

List of studies, associated focal taxonomic groups and range of correlation coefficients (rounded to two decimal places) that were used in the meta-analysis.

The meta-analysis supported our hypothesis, whereby effect sizes synthesizing the strength in cross-taxon congruence of species richness among studies decreased as the ratio of body sizes increased (Fig. 2A, R ^2^_adj_ = 0.94, p = 0.02). Overall, we found modest and positive effect sizes in the categories reflecting similar body size ratios. Specifically, our results show that body size ratios of 1∶1 and 1∶10 are significantly different from zero whereas, body sizes 1∶100 and 1∶1000–10 000 are not. A high fail safe number is associated to this analysis (Rosenthal’s n = 10 951), reflecting that our results are unlikely to change with additional research. The statistically-reduced meta-analysis that equalized the pool of studies across the body size gradient revealed a similar decrease in effect size with increasing body size ratios (Fig. 2B R^2^_adj_ = 0.97, p = 0.01) but the size of the 95% confidence intervals were more consistent across groupings. Indeed, the effect size of the full and statistically reduced meta-analyses are highly correlated (r = 1.00, p = 0.001).

## Discussion

Over the past decade, there has been a concerted effort to quantify cross-taxon congruence in inland waters across the Northern Hemisphere. Although the strength of cross-taxon congruence is variable across studies and among organismal groups, we found strong support for our hypothesis that body size is a significant predictor for the strength of species richness correlations between freshwater communities. However, similar to many other meta-analysis studies in evolution and ecology [67], our effect sizes are modest and thus further consideration of functional traits is needed. This finding has key implications for both applied and basic biodiversity questions such as the use of indicator groups and the development of predictive biodiversity models.

The results from our meta-analysis demonstrate that body size is a strong predictor for the congruence of freshwater taxonomic groups. Although our study has helped elucidate a pattern in the strength of cross-taxon congruence, body size itself is not the underlying process, but rather a commonly-used functional trait. In aquatic ecosystems, size correlates with a suite of life history traits such as metabolic rate [68], trophic level [69], survival, reproductive rate, growth and development [70]. All of these traits contribute to defining a taxonomic group’s ecological niche. Organisms most similar in size tend to occupy similar niches within aquatic ecosystems [19] and their community composition is thus driven by similar biotic and abiotic factors.

Correlations in species richness responses are believed to predominantly arise owing to common responses to environmental conditions [71], [72], [73]. In aquatic cross-taxon congruence studies, biodiversity indicators are often measured along environmental gradients such as lake area (e.g. {27,58]), acidity (e.g. [55], [60]), nutrients (e.g. [12], [55]) and habitat structural complexity (e.g. [55], [57]). Thus, organisms of the same size may have higher cross-taxon congruence due to similar life history traits that dictate similar biodiversity responses to environmental gradients. However, the relationship between the strength of cross-taxon congruence and body size does not appear to hold true across all ecosystems. A previous meta-analysis of the terrestrial literature found no effect of trophic position (a correlate of body size) on the success of cross-taxon congruence [74]. This discrepancy could, however, be due to weaker correlations between body size and trophic position in terrestrial ecosystems [75]. Although it has yet to be tested, we predict that differences in body size would contribute to the strength of cross-taxon congruence in marine ecosystems where spatial scale and habitat were previously found to be important predictors [76].

An important caveat of this meta-analysis (and all studies focused on species richness) is that species richness estimates of taxonomic groups are dependent on sample size [77] and thus may influence the results reported. However, over 70% of studies included in our meta-analysis have indeed considered sample size through the identification of individuals using standardized protocols (e.g. [12], [64]) or through rarefaction analyses (e.g. [26], [62]). Furthermore, one of the larger studies (n = 84) included in our analysis [26], quantified the variability in species richness estimates among replicates and used this to calculate estimates of maximum potential correlations in richness measurements between taxonomic groups. These maximum potential estimates were in fact higher (0.32< r <0.85) than those based on point estimates (−0.07< r <0.37). Although this type of analysis was only done on one of the studies included in our meta-analysis, these results suggest that with more within-site replication, effect sizes synthesized herein might have even been larger.

As was recently highlighted in perspective piece by Lindenmayer and Likens [78], there is a strong need to quantify the taxonomic, spatial and temporal bounds for which biodiversity surrogate relationships hold (or not). Here, we show that cross-taxon congruency is strongest with organisms most similar in size. However, the effect size for organisms of similar size is still modest (using Cohen’s criteria for interpreting effect sizes). Given that functional diversity metrics tend to provide improved predictive power over species richness metrics for numerous environmental gradients [79], we suggest that consideration of other traits could help in further refining the selection of biodiversity indicators. We propose that once additional studies become available, a meta-analysis should be conducted to consider congruence among organismal groups along multiple trait axes such as body size and active vs. passive dispersing organisms.

In addition to improving the search for biodiversity indicators, evaluating the effect of body size on the similarity of species richness patterns sheds light on the use of model organisms in basic ecological research. Undoubtedly, microorganisms have figured prominently in biodiversity-ecosystem functioning experiments, which have led to the development of consensus statements regarding the importance of biodiversity for humanity [80]. However, recent studies have shown that community structure of microorganisms and macroorganisms across the landscape is different due to variations in body size and diverging dispersal abilities [29], [81]. Our results complement these conclusions and provide a predictive framework highlighting that body size should be used as a guiding principle when drawing inferences of biodiversity patterns across organismal groups.
